# Poly-ε-caprolactone Nanoparticles Loaded with 4-Nerolidylcatechol (4-NC) for Growth Inhibition of *Microsporum canis*

**DOI:** 10.3390/antibiotics9120894

**Published:** 2020-12-11

**Authors:** Vanessa Raquel Greatti, Fernando Oda, Rodrigo Sorrechia, Bárbara Regina Kapp, Carolina Manzato Seraphim, Ana Carolina Villas Bôas Weckwerth, Marlus Chorilli, Patrícia Bento Da Silva, Josimar O. Eloy, Marcelo J. Kogan, Javier O. Morales, Rosemeire Cristina Linhari Rodrigues Pietro

**Affiliations:** 1 Department of Drugs and Medicines, School of Pharmaceutical Sciences, School of Pharmaceutical Sciences of São Paulo State University (UNESP), Rodovia Araraquara-Jaú, Km 1, Araraquara, SP 14800-903, Brazil; vanessagreatti@hotmail.com (V.R.G.); Fernando.oda@unesp.br (F.O.); rodrigo.sorrechia@unesp.br (R.S.); barbara.kapp@unesp.br (B.R.K.); camanzato@hotmail.com (C.M.S.); marlus.chorilli@unesp.br (M.C.); patrbent@yahoo.com.br (P.B.D.S.); 2 Department of Mycology, Lauro de Souza Lima Institute, Bauru, SP 17034-971, Brazil; anaweck@terra.com.br; 3 Department of Pharmacy, School of Pharmacy, Dentistry and Nursing, Federal University of Ceará, University Avenue, 2853 Rodolfo Teófilo, Fortaleza, CE 60430160, Brazil; josimar.eloy@ufc.br; 4 School of Chemical and Pharmaceutical Sciences, University of Chile, Santos Dumont 964, Independencia, Santiago 8380492, Chile; mkogan@ciq.uchile.cl (M.J.K.); jomorales@ciq.uchile.cl (J.O.M.); 5 Advanced Center for Chronic Diseases, Sergio Livingstone 1007, Santiago 8380492, Chile

**Keywords:** nanoparticles, 4-Nerolidylcatechol, antifungals, *Microsporum canis*, polycaprolactone, nanoprecipitation

## Abstract

Dermatophyte fungal infections are difficult to treat because they need long-term treatments. 4-Nerolidylcatechol (4-NC) is a compound found in *Piper umbellatum* that has been reported to demonstrate significant antifungal activity, but is easily oxidizable. Due to this characteristic, the incorporation in nanostructured systems represents a strategy to guarantee the compound’s stability compared to the isolated form and the possibility of improving antifungal activity. The objective of this study was to incorporate 4-NC into polymeric nanoparticles to evaluate, in vitro and in vivo, the growth inhibition of *Microsporum canis*. 4-NC was isolated from fresh leaves of *P. umbellatum,* and polymer nanoparticles of polycaprolactone were developed by nanoprecipitation using a 1:5 weight ratio (drug:polymer). Nanoparticles exhibited excellent encapsulation efficiency, and the antifungal activity was observed in nanoparticles with 4-NC incorporated. Polymeric nanoparticles can be a strategy employed for decreased cytotoxicity, increasing the stability and solubility of substances, as well as improving the efficacy of 4-NC.

## 1. Introduction

Dermatophyte fungal infections affect keratinized tissues and are difficult to combat. The long period of treatment, in addition to the adverse effects caused by systemic antifungal drugs, frequently leads to poor adherence to medication, favoring refractory disease [1,2]. The mycoses caused by *Microsporum canis* are more frequent in dogs and cats, but can extend the contamination to humans due to the contact between them since the pets are present inside the houses and inserted into the family.

4-Nerolidylcatechol (4-NC) is the major compound of the plant *Piper umbellatum*, popularly known in Brazil as pariparoba or caapeba. The molecule is an unstable natural product [3], and the extracts of this plant have demonstrated important biological properties, photoprotective action, leishmanicide, anti-inflammatory, anti-ulcer, anticancer, and antimicrobial activities [4,5,6,7,8]. Studies have demonstrated the high antifungal potential of 4-NC against dermatophyte fungi [9], which has motivated interest to develop this study.

4-NC belongs to the sesquiterpenes class and it is considered practically insoluble in water (solubility of −4303 (log mol/L)) [10]. The mechanism for antimicrobial action is not yet well defined, but a study with derivatives of this compound evaluating antimalarial activity showed mechanisms such as inhibition of isoprenoid biosynthesis and inhibition of hemozoin formation [11]. In human cells, it has a proteasome inhibition mechanism, with the accumulation of ubiquitinated proteins, damage to DNA, and changes in the mitochondrial membrane’s potential. These mechanisms may possibly be related to antifungal activity since the cells are eukaryotic [12].

The drug’s high antioxidant activity requires attention regarding its stability, and the lipophilic characteristic causes certain difficulties in its incorporation into formulations. To overcome these problems, the development of nanostructured delivery systems can be a viable strategy. Nanoparticles are colloidal systems, have nanometric size, and because of this, have the ability to permeate barriers in the body and protect molecules of interest, giving the incorporated molecule a higher solubility. They are acceptable for loading of both lipophilic and hydrophilic drugs [13,14].

Several polymers, either natural or synthetic, can be used for drug encapsulation. Regarding synthetic polymers, there are biodegradable aliphatic polyesters such as polylactides (PLA), poly (lactide/glycoside) copolymers (PLGA), and poly (ε-caprolactone), as well as non-degradable polymers such as poly (methyl methacrylate) and polyacrylates. Naturally occurring polymers have been widely used as carriers for dermatological disorders; however, polymers of synthetic origin such as poly-ε-caprolactone (PCL) have the advantage of higher encapsulation efficiency, purity, and high reproducibility, being more suitable for molecules with non-polar characteristics [15]. Studies using PCL showed low toxicity and compatibility with many drugs, as well as excellent solubility in organic solvents, heat stability, and good permeability, in addition to allowing slow release [16,17]. However, to the best of our knowledge, PCL has not been previously employed for 4-NC encapsulation.

Polymeric nanoparticles exhibit versatility in their structures, which can be modulated to protect/carry molecules to the action site or respond to physiological or external stimuli. Hydroxypropyl-ß-cyclodextrin (HP-ß-CD), which is a well-known membrane protector, has been shown to increase liposome solubility with 4-Nerolidylcatechol [18].

The mode of incorporation can modulate the response of the nanoparticles in biological environments under different pH conditions, as well as enzymatic, oxidative, reducing, temperature variation, and irradiation [19]. The properties and characteristics of nanostructured systems allow its use for several objectives, such as for mycoses.

Nanotechnology is an interesting strategy to improve the efficacy of traditional antifungals, reduce toxicity, improve biodistribution and drug targeting, and bring promising results in in-vitro and in vivo studies. In view of this, plant extracts can benefit from the association of this technology [20]. Liposomes containing 4-Nerolidylcatechol showed a controlled release, and modulation of hemocompatibility, in addition to a higher biocompatibility in the formulation providing stability and solubility [21].

The polymer PCL (polycaprolactone) has been successfully used for drug delivery, evaluated both in vitro and in vivo. After demonstrating in vivo biocompatibility and efficacy, PCL-based formulations have been approved by the FDA [22]. Furthermore, the reproducible production of biocompatible medical devices, including in the nanoscale, can employ the electrospinning process. For instance, Tammaro et al. (2015) [23] prepared linesolid loaded in electrospun PCL fibers for topically controlled drug delivery to inhibit *Staphylococcus aureus*. Wang et al. (2020) prepared PCL-based nanofibers for delivery of O-D-glucopyranosyl-L-ascorbic acid and heparin to treat inflammation and thrombosis [24].

This study aimed to develop polymeric nanoparticles containing 4-NC for growth inhibition of *M. canis*, evaluating the activity in vitro and in vivo.

## 2. Materials and Methods

### 2.1. 4-NC Isolation

The plant material was collected in the Horticultural Medicinal and Toxic Plants “Profa. Dra. Célia Cebrian Araujo Reis”, School of Pharmaceutical Sciences, UNESP-Araraquara, and identified and authenticated in the Laboratory of Botany of the School of Pharmaceutical Sciences—UNESP, Araraquara, Brazil. Isolation of 4-NC was performed as described by Iwamoto et al. (2015) [7], with adaptations. The fresh leaves were extracted by maceration with dichloromethane (used proportion of plant material and solvent was 0.25 Kg/L) at room temperature. The extract was filtered and evaporated to remove the solvent completely. After evaporation of the solvent, liquid/liquid was partitioned with acetonitrile/hexane (1:1). Then the acetonitrile phase was subjected to solvent evaporation for column separation. The column separation was made using a 2 cm/2 cm glass column, with C18 (octadecylsilane) as the stationary phase, and ACN:H_2_O (50:50) as the mobile phase.The isolation was confirmed by high-performance liquid chromatography and infrared spectroscopy. The analysis of 4-NC by NMR was performed with a Fourier 300 spectrometer, capable of obtaining ^1^H and ^13^C nuclei. For the infrared analysis, a Bruker^®^ Spectrophotometer (Billerica, MA, USA) model IV-FT—Alpha Platinum ATR was used, and the data were treated with OriginPro 9 SR2. The application was made directly, without dilution.

### 2.2. Development of Nanoparticles

The nanoparticles were developed by the classical nanoprecipitation methodology, using a 1:5 weight ratio (drug:polymer). The polycaprolactone polymer and 4-NC were both dissolved in 50 mL of acetone, and the Poloxamer 407 surfactant was dissolved in 100 mL of deionized water (0.5%), the aqueous phase. The organic phase was dipped into the aqueous phase with stirring in a laminar flow hood for approximately 16 h for total evaporation of the organic solvent. The final volume was 50 mL [25].

### 2.3. Encapsulation Efficiency

The experiment was performed in the laboratory of Drug Delivery at the University of Chile (UCHILE). The high-performance liquid chromatography was performed using a Perkin–Elmer Flexar apparatus, analytical mode with quaternary pumping system, equipped with a Rheodyne 6-way automatic injector with 20uL loop, PDA detector (photodiode array), and degasser. A C-18 (250 × 4.6 × 5) GL Sciences column was used, and mobile phase composed of a gradient of acetonitrile: water with 0.1% trifluroacetic acid (30:70) for 10 min, (5:95) for 15 min, flow 1 mL/min, 20 μL of injection was used. Data acquisition and processing were performed using Chromera^®^ CD N5188000-T, version 4.1.0 (Perkin–Elmer, Waltham, MA, USA) software.

To evaluate the encapsulation efficiency (EE%), an indirect method based on ultrafiltration, was employed. For this purpose, the free 4-NC was separated from 4-NC-loaded nanoparticles after centrifugation (Centrifuge 5804 R, Eppendof, Hamburg, Germany) performed in Amicon^®^ tubes for 20 min at 3800× *g* at the temperature of 26 ± 1 °C. Then the filtrate was directly chromatographed and the nanoparticles retained in the filter were suspended in 5 mL of acetonitrile to dissolve the particles, sonicated for 5 min, filtered (0.45 µm, PTFE). The efficiency was calculated based on the calibration curve performed, and followed the equation [25].

(1) $$\mathrm{EE}\%=\frac{\mathrm{ultracentrifuged}\;\mathrm{pellet}\left(\mu g\right)-\mathrm{supernatant}\left(\mu g\right)\mathrm{drug}\;}{\mathrm{theoretical}\;\mathrm{mass}\;\mathrm{of}\;\mathrm{the}\;“\mathrm{drug}”}\times 100$$

### 2.4. Dynamic Light Scattering (DLS) and Zeta Potential (Zeta)

The mean size and zeta potential of nanoparticles were measured by dynamic light scattering (DLS) using a Zetasizer Nano ZS (Malvern Instruments Ltd., Malvern, Worcestershire, UK). The samples were applied without dilution. Measurements were made at 25 °C with a fixed scattering angle of 173°, the refractive index (RI) at 1.330, and absorbance at 0.01. All analyses were performed in triplicate (*n* = 3), and the data were expressed as the mean values and standard deviations.

### 2.5. Nano Tracking Analysis (NTA)

The nanoparticles were also analyzed by nano tracking analysis (NTA), a complementary technique to DLS. The samples were applied directly on the NanoSight NS300 (Malvern Instruments Ltd., Malvern, UK). The software traces several particles individually under Brownian motion and relates the velocity of particle movement, and calculates their hydrodynamic diameters using the Stokes-Einstein equation [26]. The samples were diluted 2000 times with ultrapure distilled water, and results were expressed in particle size (nm) and particle concentration.

### 2.6. Scanning Electron Microscopy (SEM)

For the measurement in scanning electron microscopy (SEM), the nanoparticles were deposited on the copper grid. The grids were in contact with the samples for 5 min, and then they were placed in contact with water for 1 min. After this time, it was put in contact with 0.5% phosphotungstic acid for another 1 min, and finally, another 1 min with water. The preparation was performed 24 h before the analysis; the grids were kept in filter paper-lined plates and capped. The measurements were performed using a microscope FEI model Inspect F50 [27].

### 2.7. Fourier Transformed Infrared Spectroscopy (FTIR)

The FT-IR spectral studies were carried on IR-Prestige 21 Shimadzu using the KBr pellets method (hydraulic press Shimadzu, Japan ), from 400 to 4000 cm^−1^. The FTIR spectra were performed for: 4-NC on two different quantities (65 µg and 676 µg), poloxamer 407, polycaprolactone, lyophilized empty nanoparticles and lyophilized 4-NC nanoparticles.

### 2.8. In Vitro Evaluation of 4-NC and 4-NCNP Antifungal Activity

The antifungal activity was evaluated according to M38-A2 guideline with some modifications [28]. The microdilution technique used 96-well plates with RPMI medium, using a clinical isolate of *M. canis*, isolated from a dog. The concentration of 4-NC and nanoparticles with 4-NC (4-NCNP) samples ranged from 250 μg/mL to 0.244 μg/mL and 150 μg/mL to 0.146 μg/mL, respectively. Amphotericin B and terbinafine were used as controls. The inoculum concentration was 5.0 × 10^4^ CFU/mL. The plates were incubated for seven days under constant stirring at 28 °C. Minimal inhibitory concentration (MIC) was defined as the lowest concentration where growth inhibition occurred. Plates were read using 0.1% resazurin (30 μL), and minimal fungicidal activity (MFC) was performed using a sample of each well plated in PDA (potato dextrose agar) before the addition of resazurin. The plates were incubated for seven days at 28 °C. The minimal fungicidal concentration (MFC) was defined as the lowest concentration at which no growth occurred.

### 2.9. Preparation of Gel Formulations for Use in In Vivo Study

Formulations were developed to facilitate cutaneous application, so the samples were inserted into hydroxyethylcellulose gel. The base gel was prepared at the concentration of 2% in water under heating and stirring, and after cooling the samples were added. The concentration of nanoparticles used for the in vivo test was eight times the value obtained in the MIC test (0.072 mg/mL), according to the methodology used by Sharma et al. (2011) [29]. Terbinafine was used as eight times the value obtained in MIC test (0.005 mg/mL).

### 2.10. In Vivo Antimycotic Evaluation in SWISS MICE

The study was approved by the Research Ethics Committee (protocol CEUA/FCF/CAr: 06/2017). Twenty-four adult male Swiss mice weighing between 25 and 35 g were used, kept in individual cages, with feed and water at will. They were divided into four groups of six animals: infected with *M. canis*, treated with gel containing 4-NC nanoparticles, free terbinafine gel (control drug), gel with empty nanoparticle, and the negative control without treatment.

The animals (*n* = 6) were immunosuppressed with 500 μg of estradiol valerate in subcutaneous injection. After three days of immunosuppression, the infection was induced through slots with sterile needles to facilitate infection, where a suspension of 1 × 10^6^ CFU/mL of *M. canis* (clinical isolate) was applied. After the period of infection (about seven days) the treatment was started [29].

Samples treatments were applied using sterile swabs with an amount sufficient to fill the entire infected area (0.3 g). After three days of treatment, three animals from each group were euthanized. After the seven days period, the others were euthanized. Before the sacrifice, blood samples from the animals were collected through the submandibular vein [30] to evaluate the enzymes aspartate aminotransferase (AST) and alanine aminotransferase (ALT) [31]. These enzymes were evaluated to verify if there was liver damage. The areas of the infected and treated skins were collected for microscopic analysis and stored in Karnovisky’s solution for 24 h, after which time they were transferred to phosphate buffer (pH 7.4). The experimental model used is due to the greater susceptibility to dermatophytes.

### 2.11. Analysis of the Hepatic Profile of the Animals

Blood samples were centrifuged for 15 min at 1100 × *g*. After separation, the serum was transferred to 1.5 mL Eppendorf tubes and stored at −15 °C. For the analysis, the samples were removed from refrigeration and left at room temperature. Labtest^®^ AST and ALT kits were used, and the tests were performed in triplicate.

### 2.12. Grocott-Gomori Silver Methanamine Staining Microscopy

The analysis of microscopy with Grocott–Gomori staining was carried out in the laboratory of Lauro de Souza Lima Research Institute (Bauru/SP). The skins were stocked in phosphate buffer (pH 7.5) and placed in an OMA DM40 histotechnical model to dehydrate and incorporate into the paraffin. After the skins were embedded, they were cut with a four micron MRP2015 microtome. The dewaxing was started with distilled water and then subjected to chromic acid (4%) for thirty minutes, after which time it was washed with common water. Then, sodium bisulfite (1%) was added for one minute, followed by ordinary water for 10 min and finally in distilled water. Samples were placed in a greenhouse in contact with the silver methenamine solution for 30 min. After this period, the samples were washed again with distilled water six times, followed by gold chloride (1%) for 4 min, and distilled water. Sodium thiosulphate (2%) was placed for 4 min, washed in running water and ending with light green for 45 s. Finally, the samples were dehydrated with 95 °C ethanol, absolute ethanol, and xylol. The samples were observed in an Axioplan 2 microscope, model HBO50.

## 3. Results

### 3.1. 4-NC Structural Determination

**Figure 1:**
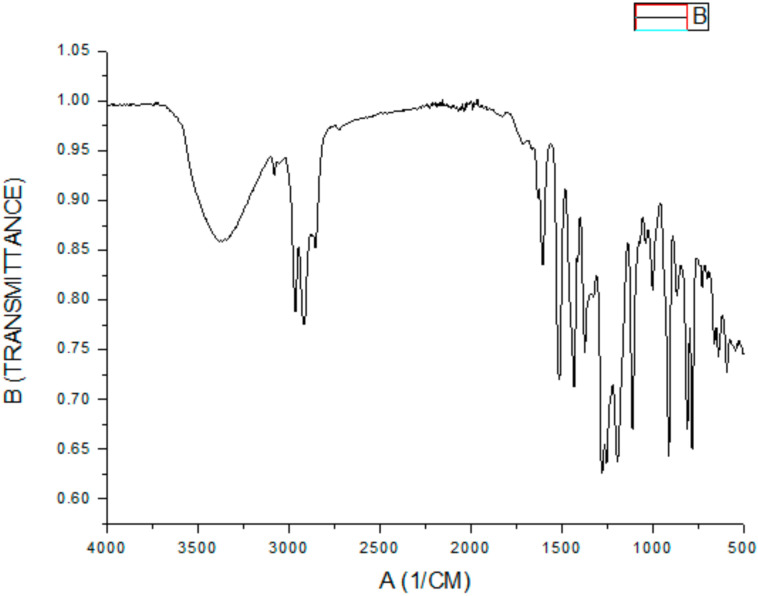
4-Nerolidylcatechol infrared spectrum.

**Figure 2:**
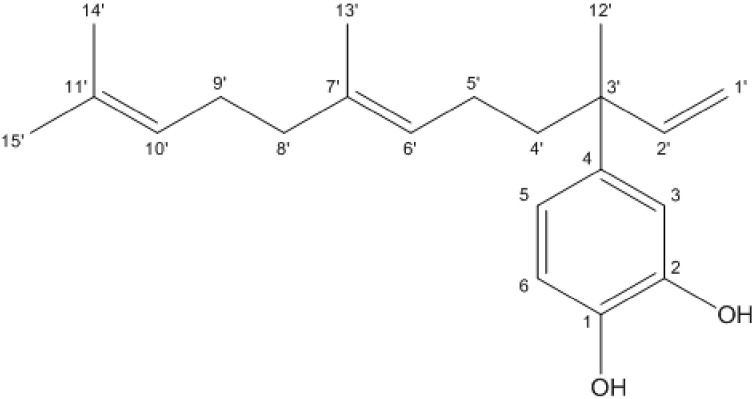
Chemical structure 4-Nerolidylcatechol.

The results obtained by the ^1^H and ^13^C spectra of 4-NC were compared to the literature for structural determination of the substance [32]. NMR spectroscopic data obtained for 4-Nerolidylcatechol at 300 MHz for ^1^H at 75 MHz for ^13^C in chloroform-d. Carbons with δ between 110 and 120 ppm are typical of aromatic rings. Thus, the C-3, C-5, and C-6 carbons were assigned to the signals in δ 115.1; 119.2, and 114.3, respectively. Other signals obtained in the ^13^C spectrum in δ 141.4 and 143.2 were attributed to the substituted carbons of the C-1 and C-2 aromatic ring, respectively, which are characteristic of the catechol group. The C-4 at δ 141.0 indicated that it was bound to the nerolidol group, which was identified by the existence of unsaturated aliphatic grouping signals, in particular by the two signals of two methylene carbons (C-5′ and C-9′) bound to the double bond methyls (δ 23.1 and 26.8, respectively) and three other vinyl methyl (C-12′, C-13′, and C-14 ′). IR spectroscopy showed high intensity in the absorbing bands with a wave number of 3400 cm-1 relative to the axial deformation of free hydroxyls (OH), also showing bands in 1635 and 1522 cm-1, indicating the presence of axial deformation of alkenes (C=C). In addition to an absorption band, 1283 cm-1 related to phenolic axial deformation (C-O) and the presence of methylene groups due to the presence of absorption bands at 3000 cm^−1^ (axial deformation C-H) (Figure 1) signals corroborated the structure of 4-NC (Figure 2).

### 3.2. Encapsulation Efficiency

**Figure 3:**
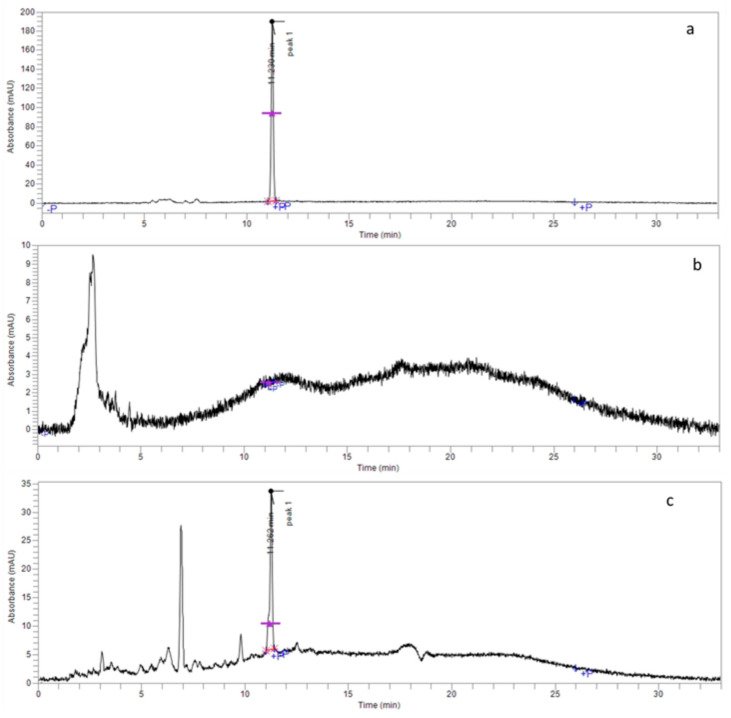
4-NC Encapsulation efficiency in nanoparticles. (**a**) 4-NC Chromatographic profile. (**b**) Chromatographic profile of 4-NC Nanoparticles filtered in Amicon^®^ tube. (**c**) Chromatographic profile of 4-NC nanoparticles retained in the Amicon^®^ tube.

**Figure 4:**
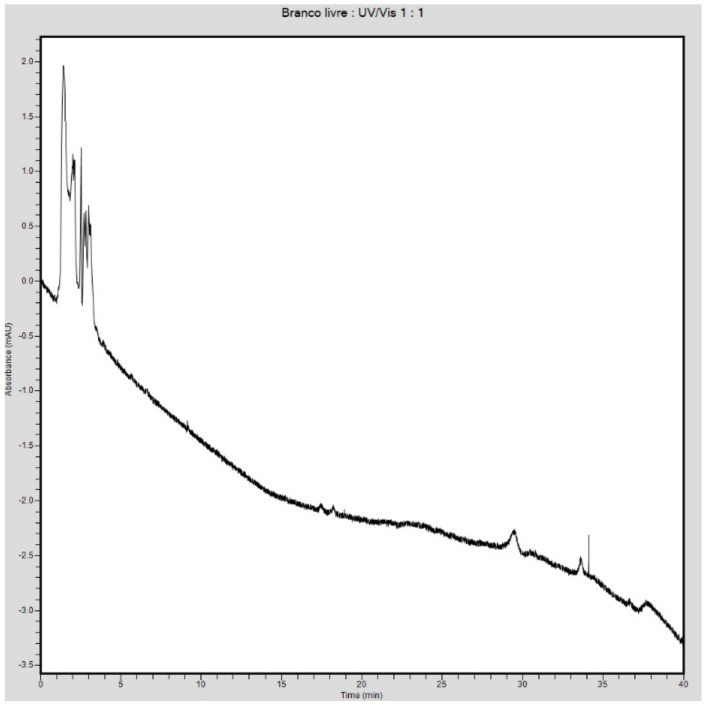
Chromatographic profile of empty nanoparticles.

The evaluation of encapsulation efficiency demonstrated 100% encapsulation efficiency in 4-NC encapsulation in polymer nanoparticles, as observed by the 4-NC profile (Figure 3a). Chromatograms obtained after centrifugation demonstrated the absence of a peak of the molecule at 11.238 min in the Amicom^®^ filtrate (Figure 3b), indicating that there was no free soluble drug. In the chromatogram of material retained in the filter (Figure 3c), the 4-NC peak was observed, attributed to the encapsulated drug. The chromatographic profile of the empty nanoparticles is shown in Figure 4.

### 3.3. DLS, Zeta Potential and NTA Characterization

The polymeric nanoparticle’s (NPs) size was 148.1 ± 1.12 nm for empty nanoparticles and 143.5 ± 1.36 nm for nanoparticles with 4-NC. The poly dispersive index (pdi) was 0.149 ± 0.01, with zeta potential (zeta) of −7.15 ± 0.16 mV for empty NPs, while nanoparticles with 4-NC showed polydispersity index of 0.232 ± 0.00 and zeta potential of −9.30 ± 0.17 mV (Table 1).

**Figure 5:**
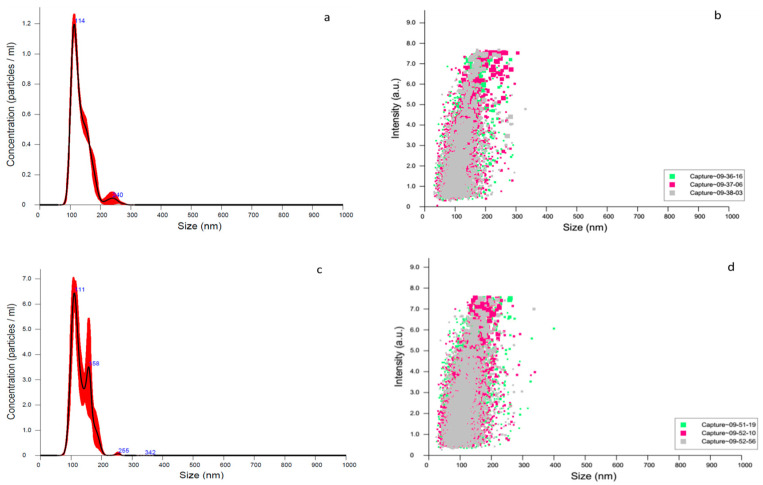
Particle size distribution. (**a**) The concentration of empty nanoparticles. (**b**) The distribution of empty nanoparticles. (**c**) The concentration of 4-NC nanoparticle. (**d**) The distribution of 4-NC.

The nanoparticles were also characterized by nano tracking analysis (NTA), showing a concentration of empty nanoparticles of 6.14 × 10^11^ ±7.74 × 10^9^ particles/mL, and nanoparticles with 4-NC demonstrated a concentration of 3.38 × 10^9^ ± 2.4 × 10^8^ particles/mL (Figure 5).

**Figure 6:**
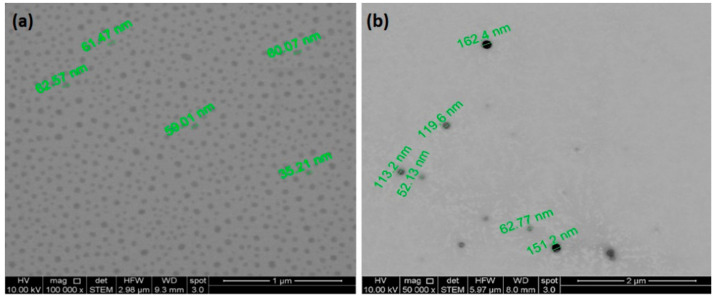
Scanning Electron Microscopy. (**a**) Empty NPs without dilution. (**b**) 4-NC NPs with 10x dilution.

The photomicrography of nanoparticles obtained for SEM showed a diameter between 35.2 and 162.4 nm (Figure 6).

### 3.4. FT-IR Spectral of Nanoparticles and Compounds

**Figure 7:**
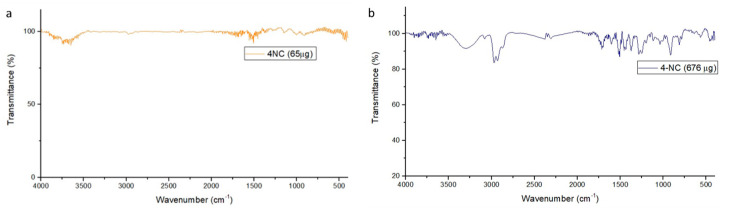
FT-IR spectra of 4-NC (**a**) 65 µg (**b**) 676 µg.

The FT-IR for 4-NC 65 µg did not show any stretching spectra, which could be due to the FT-IR sensibility equipment (Figure 7).

The FT-IR for the 4-NC 676 µg shows high-intensity bands on 3300 cm^−1^ associated to free –OH groups stretching vibrations and strong and broad signal. Other signals were shown on 2900 cm^−1^ related to C-H alkane vibrations. Besides, the 4-NC have more characteristics on 1700 and 1600 cm^−1^ related to alkenes C=C oleafinic and C-C bonds on aromatics, 1200 cm^−1^ the C-O phenolic stretch, and 900 cm^−1^ the C-H alkene oleafinic vibrations.

**Figure 8:**
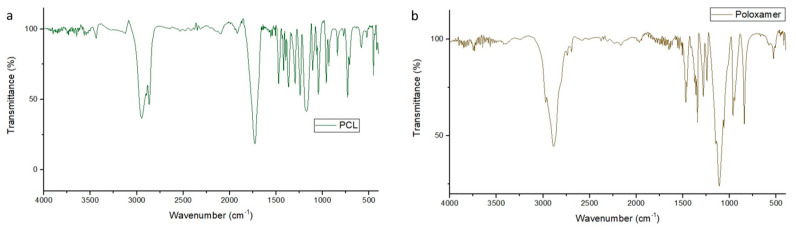
FT-IR spectra. (**a**) PCL, (**b**) poloxamer.

PCL spectra showed on 3000 cm^−1^ C-H alkane vibrations, 1700 cm^−1^ a strong narrow signal expected from the carbonyls and C-O bonds stretching on 1300–1100 cm^−1^ (Figure 8a). The IR spectrum of poloxamer 407 shows the absorption peaks at 2800 cm^−1^ related to C-H alkane stretch, a signal on 1300–1100 cm^−1^ associated with C-O ether stretch vibration (Figure 8b).

**Figure 9:**
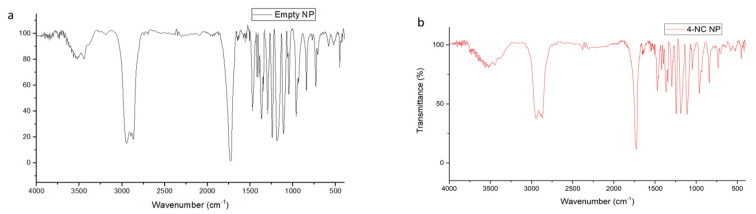
FT-IR spectra for NP. (**a**) Empty, (**b**) with 4-NC loaded.

The empty nanoparticles showed a residual water signal at 3500 cm^−1^, a signal at 2800 cm^−1^ related to the C-H alkane bonds to PCL and Poloxamer 407, a strong narrow signal at 1700 cm^−1^ associated to the carbonyl vibrations of the PCL, and a shorter signal on the 1100 cm^−1^ that corresponded to the C-O ether bond of the Poloxamer 407, indicating an interaction with the PCL (Figure 9).

**Figure 10:**
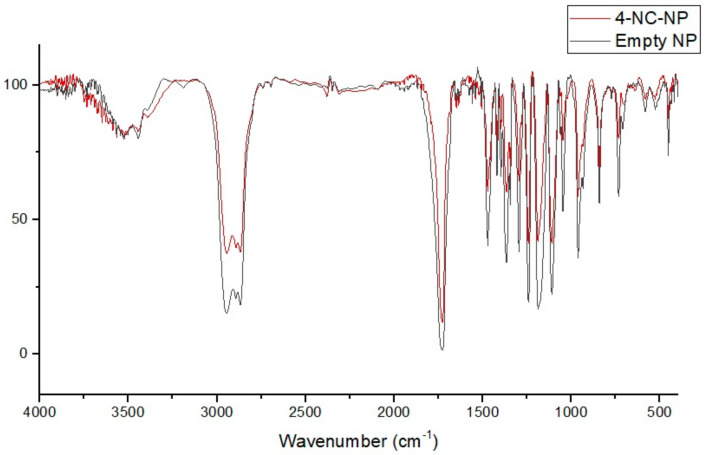
Comparison of 4-NC NP and empty NP FT-IR spectrum.

The incorporated nanoparticles with 4-NC also showed a band assigned to the vibrational mode νOH at 3500 cm^−1^, characteristic of the presence of water, the C-H alkane bonds at 2800 cm^−1^ due to the PCL and poloxamer characteristics, the carbonyls vibration bond at 1700 cm^−1^ for the PCL molecule, and a reduced signal at 1100 cm^−1^ for the C-O ether bond. The comparison of the FTIR spectral of the 4-NC incorporated and empty nanoparticules showed that the profile of the spectra were similar, showing the water signal at 3500 cm^−1^, the C-H aliphatic bond vibrations at 2800 cm^−1^, the strong narrow band at 1700 cm^−1^ due to the carbonyl of PCL, and a reduction of the signal from 1500–900 cm^−1^ on the 4-NC-NP (Figure 10).

The spectra results showed that the equipment sensibility could show any 4-NC signal in a 65 µg concentration, and the 4-NC NP were made with 45 µg. Therefore, the 4-NC molecule did not demonstrate a different profile on nanoparticle’s signal due to the low concentration, however, the 4-NC NP showed a strong, distinct biologic activity when compared with the empty NP.

### 3.5. Nanoparticles Antifungal Activity

Table 2 shows the in vitro antifungal activity, evidencing that when the drug was nanoencapsulated, there was an increase of 9.61 times in the MIC and 19.23 times for the MFC. Terbinafine and amphotericin B, control drugs, were evaluated only in free form, without nanoencapsulation.

### 3.6. In Vivo Analysis

**Figure 11:**
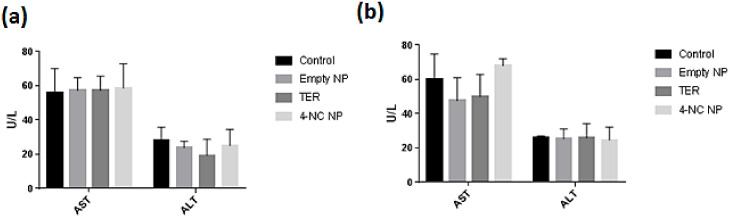
Analysis of the alanine aminotransferase (ALT) and aspartate aminotransferase (AST) enzymes from treated and untreated animals. (**a**) Control, no treatment; TER, free terbinafine; 4-NC NP, Nanoparticles with 4-NC; Empty NP, empty nanoparticles, with 3 days treatment. (**b**) Control, no treatment; TER, free terbinafine; 4-NC NP, nanoparticles with 4-NC; Empty NP, empty nanoparticles, with 7 days treatment. The statistical analysis was performed by Graph Pad Prism Version 7.0 software, through analysis of variance (ANOVA) and Dunnett’s test, establishing *p* <0.05 as the significance level.

The results showed that there were no changes in liver enzymes resulting from the treatments (Figure 11).

In assessing the potential damage to the liver, the levels of transaminases were checked in the plasma, and no significant variation in liver enzymes was observed during the period of 3 and 7 days of treatment. There were evaluated using analysis of variance (ANOVA) and Dunnett’s test, establishing a *p*-value <0.05 as the significant level. No statistically significant difference was observed between the drug-treated and control groups.

**Figure 12:**
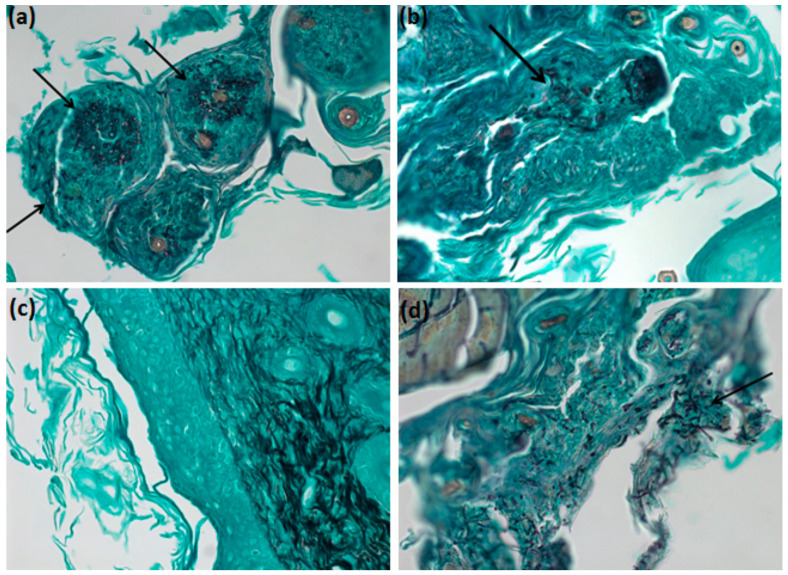
Grocott–Gomori microscopy of the skins of the euthanized animals with three days of treatment. (**a**) The skin of an animal infected with *M. canis* and without treatment. (**b**) The skin of an animal treated with 4-NC nanoparticles. (**c**) The skin of an animal treated with terbinafine. (**d**) The skin of an animal treated with empty nanoparticles.

The silver methenamine staining of Grocott–Gomori, widely used in fungal detection histology, showed *M. canis* hyphae around the hair follicles indicated by the arrows (Figure 12a), when the skin of an infected with *M. canis* and untreated animal, euthanized three days post-infection was observed. The euthanized animals with 7 days of treatment did not present any more infections, suggesting a recomposition of their immunity. Free terbinafine, after three days of treatment, showed no evidence of *M. canis* infection.

## 4. Discussion

The identification of 4-NC was carried out by nuclear magnetic resonance of hydrogens and carbons. 4-NC shows characteristic signs of aromatic and aliphatic hydrogens. Signals in the region between δ 5 and 6 (ppm) are usually attributed to olefins, whereby the signals in δ 5.02 and 5.98 were respectively assigned to the hydrogens bound to the C-1′ and C-2′, and the hydrogens connected to the C-6′ and C-10′ carbons. Signals in the region between δ 1 and 2 (ppm) correspond to methyl, so we can see in δ 1.33 (3H), δ 1.68 (3H), 1.51 (3H), and δ 1.59 (3H), with a chemical shift in the last three signals confirming the presence of double bond, these signals are attributed to the hydrogens connected to the C-12′, C-14′, C-15′, and C-13′ carbons, respectively. The signals corresponding to the aromatic hydrogens presented chemical displacement at δ 6.84, δ 6.74, and at δ 6.79, confirming the catechol grouping. The signals obtained from carbons at δ 141.4 and δ 143.2 are characteristic of the catechol system, corresponding to carbons C-1 and C-2. The ^1^H NMR and ^13^C NMR signals are in agreement with data obtained in the literature. The molecular weight of the 4-NC obtained was 314.46 g, the chemical formula being C_12_H_30_O_2_. These results are similar to the results found by Iwamoto et al. (2014) [7], Bergamo (2003) [32], Almeida (2011) [33], and Freitas (2015) [34].

In this study, it was possible to observe that the 4-NC showed total encapsulation (100%), corroborating that the antimicrobial activity demonstrated was of the nanoparticles containing 4-NC. The results obtained showed encapsulation of incorporation superior to those found by Abriata et al. (2017) [25], who used the same polymer and surfactant to incorporate ursolic acid, obtaining results 36.44% lower when using in the same concentrations of compound and polymer. Using the nanoprecipitation technique for amphotericin B incorporation, the results of the encapsulation efficiency were 60 to 80% in the formulations prepared [35].

Analysis of zeta potential demonstrated that poly-ε-caprolactone nanoparticles have a negative potential. In the study by Shao et al. [36], the nanoparticles with negative zeta potential caused little damage to the cell membranes, and the authors justified this result due to the weak interactions with the cells. This low interaction with the biomembranes favors low cytotoxicity.

The characterization analysis of the nanoparticles by DLS, showed an approximate size of 143.5 to 148.1 nm, a much smaller size when compared to the nanoparticles obtained by Souza et al. (2012) [37], which incorporated the herbicide atrazine through the nanoprecipitation technique, but using the surfactant polysorbate 80 (Tween^®^ 80). They obtained diameters up to 341 nm using the same polymer (PCL), and the same technique, i.e., nanoprecipitation.

Comparing the size data obtained by DLS and NTA, it was observed that NTA presented results of smaller nanoparticles. This type of result was also observed by other authors [19,38]. NTA analysis is a complementary technique for the characterization of nanoparticles. The difference between NTA and DLS is that the first provides the concentration of the suspended nanoparticles, and is possible to visualize the sample through videos and obtaining the presented peaks with better resolution [19].

Scanning electron microscopy showed that the nanoparticles have a lower size than that determined by analysis performed in solution and through light or laser beams. This is because, in the microscopic analysis, the samples are dried on a copper grid, and in this way, the reading does not take into account loads that could be around the particle, as in the tests that are carried out in solution, such as DLS and NTA.

Perecin et al. (2016) [39] encapsulated magnetite on a PCL and poloxamer system on a 1:5 ratio magnetite and PCL. The FT-IR spectrum of the nanoparticles does not demonstrate the interaction of the incorporated nanoparticle made of PCL and poloxamer, because the magnetite spectra showed only one strong signal at 600 cm^−1^ and that signal did not appear on the incorporated nanoparticle.

Soares et al. (2009) [40] studied a 4-Nerolidylcathecol system with 2-hydroxypropyl-β-cyclodextrin on a 1:1 molar ratio and the FT-IR spectrum of the 4-NC shows similar signals to out spectra. The 4-NC incorporated into the 2-hidroxypropyl-β-ciclodextrin was not capable of a strong, significant and different signal modification even on a 1:1 molar ratio. The incorporated signal on 3400 cm^−1^ is related to O-H aromatic bond of the sugars and the 1100–1000 cm^−1^ signal due to the ether bond vibration present on the cyclodextrin.

There is a need for further studies of release kinetics in order to improve the formulation and accelerate the release process of the 4-NC of the nanoparticles so that in the free form, they can exert the expected activity in a controlled manner and guarantee the stability of 4-NC-loaded nanoparticle.

The polymeric nanoparticle without 4-NC incorporation did not demonstrate antimicrobial activity; the antifungal activity resulted from the compound incorporated. Nanoparticles with 4-NC compared to the 4-NC isolated demonstrate a higher MIC of 4-NC nanoparticles. The antifungal activity was not potentiated by the incorporation, but neither was inhibited, which could be a consequence of slow-release from nanoparticles. However, the slow release could be an advantage for long-term use of treatment, which could be further investigated in future studies.

It has already been demonstrated in the literature that 4-NC shows certain cytotoxicity for some cell strains [7], besides possessing an antioxidant characteristic [19], is an unstable compound31, and exhibits low polarity, making difficult its incorporation into aqueous formulations. Due to these difficulties, nanostructured systems are an alternative to the use of this molecule for different biological applications.

The in vivo test of liver enzyme profiles of the animals was shown to have the same standard dosage of the ALT enzyme for the treated groups compared to the untreated group, indicating the absence of toxicity. In the present study, the results for AST were significantly lower when compared to the results of the untreated group studied by Nogueira-Neto et al. (2012) [41], which obtained results of 49.67 U/L. Similar data were found by Araujo (2012) [42] comparing Swiss mice with two different breeding strains, where they observed alterations in the values of the animals without any treatment for both ALT and AST. Studies of AST performed by Branco et al. (2011) [43] showed mean values 277.0 ± 18 U/L, whereas values of the present study were significantly lower in all groups. The evaluation of liver enzymes brings promising data since they demonstrate that there was no hepatic damage resulting from the treatment, which is a concern when using 4-NC.

The mouse skins stained with silver methenamine by Grocott–Gomori staining technique showed that groups infected with *M. canis* and treated with nanoparticles, after three days of treatment, still present some infection, however in less extension than the untreated group. The terbinafine group, which was constituted of free drug, no longer had fungi in the analyzed skin areas. Although it is a qualitative analysis, it was shown that nanoparticles are promising for the treatment of filamentous fungi infection.

This study showed that 4-Nerolidylcatechol had high encapsulation efficiency when associated with the nanoprecipitation technique with poly- ε -caprolactone, allowing a more stable formulation, since the evaluation of encapsulation demonstrated that there was no degradation of the compound. The encapsulation allows a possible controlled release for antifungal treatment that is long and difficult. Adjustments to the formulation are required in order to allow the antifungal activity of the compound to be maintained at lower levels even after encapsulation.

## Figures and Tables

**Table 1:** Zeta potential, polydispersion index, and size of nanoparticles.

	Zeta Potential (mV)	pdi	Size (nm)
Empty NP	−7.15 ± 0.16	0.149 ± 0.01	148.1 ± 1.12
4-NC NP	−9.30 ± 0.17	0.232 ± 0.00	143.5 ± 1.36

Empty NP: nanoparticles without 4-NC; 4-NCNP: nanoparticles with 4-NC.

**Table 2:** Minimal inhibitory concentration (MIC) and minimal fungicide concentration (MFC) of 4-NC, nanoparticles, and controls (µg/mL).

Samples *	MIC	MFC
4-NC	7.8	7.8
NP	-	-
4-NCNP	75	150
Amphotericin B	0.5	0.5
Terbinafine	0.625	0.625

*: 4-NC, 4-Nerolidylcatechol; NP, nanoparticles; 4-NCNP, nanoparticles with 4-NC. Data are representative of three independent experiments.
